# Analysis of Whole-Genome Sequences of Infectious laryngotracheitis Virus Isolates from Poultry Flocks in Canada: Evidence of Recombination

**DOI:** 10.3390/v12111302

**Published:** 2020-11-12

**Authors:** Ana Perez Contreras, Frank van der Meer, Sylvia Checkley, Tomy Joseph, Robin King, Madhu Ravi, Delores Peters, Kevin Fonseca, Carl A. Gagnon, Chantale Provost, Davor Ojkic, Mohamed Faizal Abdul-Careem

**Affiliations:** 1 Faculty of Veterinary Medicine, University of Calgary, Health Research Innovation Center 2C53, 3330 Hospital Drive NW, Calgary, AB T2N 4N1, Canada; ana.perezcontreras@ucalgary.ca (A.P.C.); fjvander@ucalgary.ca (F.v.d.M.); slcheckl@ucalgary.ca (S.C.); 2 Animal Health Centre, Ministry of Agriculture, Abbotsford, BC V3G 2M3, Canada; tomy.joseph@gov.bc.ca; 3 Agri Food Laboratories, Alberta Agriculture and Forestry, AB T6H 4P2, Canada; blking@telus.net; 4 Animal Health and Assurance, Alberta Agriculture and Forestry, AB T6H 4P2, Canada; madhu.ravi@gov.ab.ca (M.R.); delores.peters@gov.ab.ca (D.P.); 5 Provincial Laboratory for Public Health, Calgary, AB T2N 4W4, Canada; kevin.fonseca@albertaprecisionlabs.ca; 6 Swine and poultry Infectious Diseases Research Center (CRIPA–Fonds de Recherche du Québec), Faculté de Médecine Vétérinaire, Université de Montréal, 3200 rue Sicotte, Saint-Hyacinthe, QC J2S 2M2, Canada; carl.a.gagnon@umontreal.ca (C.A.G.); chantale.provost@umontreal.ca (C.P.); 7 Laboratory, University of Guelph, Guelph, ON N1G 2W1, Canada; dojkic@uoguelph.ca

**Keywords:** herpesvirus, recombination, mutation, molecular characterization, phylogenetic analysis, poultry, vaccine

## Abstract

Infectious laryngotracheitis virus (ILTV) is a herpes virus that causes an acute respiratory disease of poultry known as infectious laryngotracheitis (ILT). Chicken embryo origin (CEO) and tissue culture origin (TCO) live attenuated vaccines are routinely used for the control of ILT. However, vaccine virus is known to revert to virulence, and it has been recently shown that ILT field viral strains can undergo recombination with vaccinal ILTV and such recombinant ILT viruses possess greater transmission and pathogenicity potential. Based on complete or partial genes of the ILTV genome, few studies genotyped ILTV strains circulating in Canada, and so far, information is scarce on whole-genome sequencing or the presence of recombination in Canadian ILTV isolates. The objective of this study was to genetically characterize the 14 ILTV isolates that originated from three provinces in Canada (Alberta, British Columbia and Quebec). To this end, a phylogenetic analysis of 50 ILTV complete genome sequences, including 14 sequences of Canadian origin, was carried out. Additional phylogenetic analysis of the unique long, unique short and inverted repeat regions of the ILTV genome was also performed. We observed that 71%, 21% and 7% of the ILTV isolates were categorized as CEO revertant, wild-type and TCO vaccine-related, respectively. The sequences were also analyzed for potential recombination events, which included evidence in the British Columbia ILTV isolate. This event involved two ILTV vaccine (CEO) strains as parental strains. Recombination analysis also identified that one ILTV isolate from Alberta as a potential parental strain for a United States origin ILTV isolate. The positions of the possible recombination breakpoints were identified. These results indicate that the ILTV wild-type strains can recombine with vaccinal strains complicating vaccine-mediated control of ILT. Further studies on the pathogenicity of these ILTV strains, including the recombinant ILTV isolate are currently ongoing.

## 1. Introduction

The infectious laryngotracheitis (ILT) was first reported in Canada in 1925 [1]. The causative virus, infectious laryngotracheitis virus (ILTV), is a member of the family *Herpesviridae*, subfamily *alphaherpesvirinae* and genus *Iltovirus*, known as *Gallid herpesvirus-1* (GaHV-1). Peafowls and pheasants are able to contract this virus, but chickens are considered the main host [2,3]. ILTV is an enveloped virus with glycoprotein projections on its surface. It contains an icosahedral nucleocapsid with linear double-stranded deoxyribonucleic acid (DNA) genome of an approximate length of 150 kilobase pairs (kbps). The genome is comprised of a Unique Long (UL) region and a Unique Sort (US) region, flanked by two inverted repeat regions, an internal repeat (IR) region and a terminal repeat region [4]. The ILTV genome contains 76 open reading frames (ORFs), from which 63 are homologous to those of the *herpes simplex virus* (HSV) *1*, 6 are *Iltovirus* genus-specific, and one ORF (UL 0) is only found in the ILTV genome [4].

ILTV is known to have tropism for the tracheal mucosa and conjunctiva, and it causes an acute upper respiratory tract disease. Presentation of clinical signs can go from mild to severe, ranging from nasal and eye secretions to conjunctivitis, reduced weight gain and decrease in egg production to the most characteristic bloody mucus expectoration, gasping and death [1]. ILTV can establish lifelong infections by establishing latency in the trachea [5] and trigeminal ganglia coinciding with the development of the adaptive immune response [6]. Latent ILTV can be periodically reactivated when the affected birds are exposed to stressful episodes, such as extreme weather conditions, nutritional imbalances, transportation, the onset of laying or relocation to a new flock [7].

Control of ILT relies largely on biosecurity measures and vaccination [8]. The commonly used vaccines for ILTV control are recombinant ILT vaccines with either herpesvirus of turkeys (HVT) or fowlpox virus (FPV) as vectors, as well as live attenuated vaccines. Based on the method of attenuation, attenuated vaccines can be categorized into either chicken embryo origin (CEO) or tissue culture origin (TCO) [9]. Attenuated vaccines are usually preferred over recombinant vaccines, given the fact that they provide fast and long-lasting protection and induce stronger cellular immunity [8]. However, vaccine viruses present in attenuated vaccines can still go into a state of latency only to be shed later by the infected birds. Attenuated vaccine virus is also prone to reversion to virulence by multiple passages through bird-to-bird transmission within the flock. This event is reported more often among CEO vaccines given the lower degree of attenuation in comparison to TCO vaccines [10,11,12].

Mutations are very rare in viral DNA genomes [13]; however, recombination among herpesviruses has been well documented and is considered to be a key evolutionary strategy [14]. Recombination can occur more frequently with the use of attenuated vaccines when coinfection with a vaccine and/or wild-type strain takes place. Furthermore, studies have provided evidence confirming recombination between ILTV strains [15,16,17], with recombination breaking points mostly identified within the UL and IR regions of the genome [18,19]. Recombination has been reported mostly among strains related to the CEO vaccines, resulting in new viruses with higher virulence than that of their parental strains [20]. Studies conducted in Australia [21], United States (US) [9], Italy [20], China [22] and Korea [23] have indicated that circulating ILTV strains were genetically related to attenuated CEO vaccine viruses, and in some cases, vaccine recombinants [24].

ILTV infection is endemic in backyard flocks in Canada, and sporadic ILT outbreaks are commonly observed in commercial flocks [25]. CEO and TCO, as well as recombinant vaccines, are currently available in Canada. For more than 10 years, backyard flock owners were urged to vaccinate their birds with TCO vaccines in some of the provinces, and it is possible that backyard flocks vaccinated with live attenuated vaccines could be infected with wild-type virus. Such situations may facilitate co-circulation of two different ILTV strains, which could potentially enable recombination, as has been seen in other countries [19]. Few studies have addressed the molecular nature of ILTV circulating in Canada using either complete or partial genes of the ILTV genome (UL 47, US 8, ORF a and ORF b). These studies have been successful in differentiating between wild-type and vaccine-related ILTV strains and have reported the presence of both CEO vaccine virus and wild-type ILTV in the Canadian chicken flocks [26,27]. However, to this date, there are neither whole-genome sequences of Canadian-origin ILTV strains nor any available information to suggest that ILTV recombination has ever occurred in Canada. The objective of the study was to genetically characterize the ILTV isolates obtained from ILTV clinical cases in Alberta (AB), Quebec (QC) and British Columbia (BC) through whole-genome sequencing (WGS) as well as to provide evidence for recombination events if present in these isolates.

## 2. Materials and Methods

### 2.1. Samples

Quantitative polymerase chain reaction (qPCR) assays targeting the ICP4 (primers, F: 5′-GGGTCGGTTCAGTCAGTAA-3′; R: 5′-GGTCATCGACCAAAGACTGT-3′ and probe 5′-GAGGTCGACGGCCAACGC-FL-3′) and glycoprotein (g) G genes (primers, F: 5′ -ATTGCCACCGTTTCCCTAG-3′; R: 5′-CCATTTCACCTCGACTGACACT-3′ and probe, 5’-CAACCGCACCACGATTGAGG-FL-3′) have been used to quantify ILTV genome in nucleic acids of tracheal swabs and tissues (*n* = 46) originated from backyard flocks of AB. A qPCR assay targeting ILTV thymidine kinase gene (primers, F: 5′- CGA GAA CGA TGA CTC CGA CTT -3′; R: 5′- GGC CCG TCG ACG TAA AGA -3′ and TaqMan probe, 5′-6-FAM-CGC CGC GTT GTA C-MGBNFQ-3′) has been used to detect ILTV genome in clinical samples (*n* = 9) originated from commercial poultry operations in BC. The qPCR reactions have been carried out using LC FastStart DNA Master Hybridization Probes Kit (Roche Diagnostics, Laval, QC, Canada) or One-Step RT–PCR Master Mix (Life Technologies Inc, Austin, TX, USA) according to manufacturers recommendation and run using LightCycler thermocycler (Roche Diagnostics GmbH, Mannheim, State of Baden-Wurttemberg, Germany) or an Applied Biosystems Inc (ABI) 7500 Fast Real-Time PCR unit (ABI, Foster City, CA, USA). The amplification conditions consisted of initial denaturation of 50 °C for 2 min followed by denaturation of 95 °C for 10 min, then 40 cycles of denaturation of 95 °C for 15 s and annealing at 60 °C for 1 min. A qPCR assay targeting gC gene (primers, F: 5′-CCTTGCGTTTGAATTTTTCTGT-3’; R: 5′-TTCGTGGGTTAGAGGTCTGT-3′ and TaqMan probe, 5′-6-FAM-CAGCTCGGTGACCCCATTCTA-BHQ1-3′) has been used to screen clinical samples (*n* = 3) originated in QC commercial operations as has been described previously [28]. These ILTV-positive samples (*n* = 58) were obtained from the Agri Food Laboratories, Alberta Agriculture and Forestry in Edmonton, AB, the Animal Health Center in Abbotsford, BC and the Laboratoire de Diagnostic Moléculaire (LDM), University of Montreal, in St-Hyacinthe, QC, respectively. In order to get enough viral DNA for WGS, the ILTV samples were propagated in either chorioallantoic membrane (CAM) of 9- to 11-day-old specific-pathogen-free (SPF) chicken embryos or monolayers of hepatocellular carcinoma cell lines (LMH) to increase virus titer. Only 14 of the samples were successfully propagated and each sample required between one to three passages before submission for WGS.

### 2.2. ILTV Propagation on CAM and LMH Cells

The use of embryos was approved by the Health Science Animal Care Committee (HSACC) of the University of Calgary (Protocol number: AC19-0013). The ILTV isolates were propagated on the CAM of SPF eggs on embryo day 10, obtained from the Canadian Food Inspection Agency (CFIA) in Ottawa, Canada.

On viable embryonated eggs, the air cell was located by candling. A small mark was placed in the eggshell 0.5 cm right below the bottom edge of the air cell. Carefully, while holding the previously disinfected egg in a horizontal position, and with the previously mentioned mark looking upwards, a puncture was made in the eggshell above the mark using a 25-gauge needle without introducing the needle too deep to avoid damaging the embryo. Another puncture was made in the shell at the top of the air cell while still maintaining the egg in a horizontal position. Using a rubber bulb covering the orifice made in the air cell, air was taken out of the air cell to create an artificial air cell on the first puncture via negative pressure [29]. The inoculum was then deposited in the artificial air cell and 5 days post-inoculation the CAM is collected from the eggs. CAMs were placed in 2 milliliter (mL) tubes containing sterile zirconium beads (Benchmark Scientific, NJ, USA), 1 mL of Dulbecco’s phosphate-buffered saline (DPBS) with 1% of penicillin–streptomycin (Gibco Life Technologies, Burlington, ON, Canada). Shortly after, the CAMs were homogenized using a microtube homogenizer Bedbug d1030 by Benchmark Scientific. The supernatant was collected, aliquoted and kept at −80 °C for further use.

For propagation of the ILTV isolates on cell monolayers, T-75 tissue culture flasks (Greiner Bio One, Kremsmünster, Austria) precoated with 2% gelatin and containing a confluent monolayer of LMH cells were used. Each flask contained 15 mL of Dulbecco’s Modified Eagle Medium (DMEM) containing 10% fetal bovine serum and 1% penicillin/streptomycin (Gibco Life Technologies, Burlington, ON, Canada). The monolayer of LMH cells was inoculated with the ILTV Canadian isolates and incubated at 37 °C in 5% CO_2_ for 72 h. After the incubation period, flasks were submitted to three freeze- (−80 °C) and thaw- (37 °C) cycles of 30 min each, after which cells and supernatant were collected and taken for ultracentrifugation on a high-speed centrifuge Avanti J-26 (Beckman Coulter Life Sciences, IN, USA) at 50,000× *g* for three hours. After ultracentrifugation, the supernatant was discarded, and the cell pellets were resuspended in 500 microliters (µL) of DMEM and aliquoted in small vials and stored at −80 °C until further use.

### 2.3. Viral DNA Purification from Homogenized LMH Cells and qPCR

DNA purification was carried out using QIAmp DNA Mini Kit (QIAGEN GmbH, Hilden, Mettmann, Germany), according to the manufacturer’s instructions, assessed for purity and quantified using a Nanodrop ND-1000 spectrophotometer (Thermo Scientific, Wilmington, DE, USA).

A quantitative PCR assay was carried out using a CFX96-c1000 Thermocycler (Bio-Rad laboratories, Mississauga, ON, Canada) targeting the proteinase K (PK) gene, as described previously [30,31]. The total volume per reaction was 20 µL, which included 20 nanograms (ng) of genomic DNA, the Fast SYBR Green Master Mix (Invitrogen, Burlington, ON, Canada), 10 µL of SYBR Green, 0.5 µL of forward and reverse specific primers with a final concentration of 10 picomolar (pmol)/ µL targeting the ILTV PK gene (F: 5′-TAC GAT GAA GCG TTC GAC TG -3′ and R: 5′-AGG CGT GAC AGT TCC AAA GT -3′) and DNAse/RNAse-free water (Thermo Scientific, Wilmington, DE, USA). Thermocycler conditions were 95 °C for 20 s for initial denaturation, then 40 cycles of denaturation to 95 °C for 3 s, annealing at 60 °C for 30 s and elongation at 95 °C for 10 s.

### 2.4. Sample Submission for WGS and Genome Reconstruction

The samples with high ILTV genomic content were directly submitted for WGS (LDM, Faculty of Veterinary Medicine, University of Montreal, QC, Canada) and sequenced using the MiSeq platform (Illumina Corp, San Diego, CA, USA). To remove Illumina adaptors, automatic adaptor trimming was selected on the MiSeq spreadsheet following CLC Genomic Workbench (QIAGEN, Redwood, CA, USA). Right before de novo assembly, another step of trimming was performed with the following settings: minus strand only (3′ end trimming); allow internal and end matches with a minimum internal score of 10 and a minimum end score at 4 and alignment scores cost (Mismatch cost at 2 and Gap cost at 3).

For quality filter, settings were set at Q30 reads. Further, using CLC Genomic Workbench Quality Trimming, with the Trim using quality scores with the limit set at 0.05, the Trim ambiguous nucleotides with a maximum number of ambiguities set at 2 and the discard reads below 15 selected.

For genome reconstruction, all reads were mapped to a complete genome ILTV GenBank reference (Supplementary Table S1) with the Map Reads to Reference tool in CLC Genomic Workbench, with no reference masking selected, match score at 1, mismatch cost at 2, linear gap cost selected, autodetect paired distance selected, and the map randomly selected in the nonspecific match handling box.

The sequence with the most and longest reads mapped against was used as the reference to perform the mapping of all reads with the same setting as described previously. A second mapping was then used to obtain the consensus sequence with the Extract Consensus Sequence tool with the options as following: low coverage definition threshold set at 5, insert N ambiguity symbols selected, in the conflict resolution vote selected, and the use quality score checked.

Consensus sequences with the Extract Consensus Sequence tool with the options were set as follows: Low coverage definition Threshold set at 5, Insert N ambiguity symbols selected, in the Conflict resolution vote selected, and the Use quality score checked. Thus, the threshold was set at 5 (Supplementary Table S1).

### 2.5. Genotyping Based on Complete ILTV Genome

For phylogenetic analysis, the obtained sequences were aligned with ILTV sequences available in the National Center for Biotechnology Information (NCBI) database. Sequences known to be a product of experimental studies on recombination were not deemed necessary to fulfill the ends of this research and hence, were not included in this study. The sequences included in this analysis mostly comprised of field strains and live attenuated CEO and TCO vaccine sequences from different geographic backgrounds (Supplementary Table S2). Multiple Sequence Alignment was done with Fast Fourier Transform (MAFFT v7.450 [32]) in Geneious^®^ v10.2.6 [33]. Sequences and alignments were visually inspected. Sites where ambiguous nucleotides were present in the AB ILTV sequences were replaced by the consensus sequence only in sites with no nucleotide variability in the consensus. Next, a phylogenetic tree of the complete sequence alignment was generated using a Bayesian inference method using MrBayes 3.2.6 [34] with default settings. It should also be mentioned that phylogenetic analysis using maximum-likelihood resulted in very similar inferences as the Bayesian inference method. Additional phylogenetic trees were constructed using the UL, US and IR regions of the ILTV genome of the 50 sequences to gather key information on the presence of recombination in any of the sequences. Posterior probability values (given as decimals) indicating the support for any given branch are displayed as branches labels in each of the phylogenetic trees.

### 2.6. Recombination Analysis

Recombination analysis and detection of crossover points in the 50 aligned genome sequences were conducted in the Recombination Detection Program (RDP4 v.4.80) [35] using default settings. Recombination events suggested by RDP4 software were confirmed by using Similarity Plotting (Simplot, 3.5.1, SCRoftware, Baltimore, MD, USA) [36], with a window size of 6000 and a step size of 200.

## 3. Results

### 3.1. Flock Background Information

qPCR-confirmed ILT-positive clinical samples originated from AB belonged to small backyard flocks, while the ILT samples originated from QC, and BC belonged to larger commercial flocks. Only two flocks had a history of ILT vaccination: one of the flocks in QC and the other one from AB. All of the relevant flock history and clinicopathological findings are summarized in Table 1 and Supplementary Table S3.

### 3.2. ILTV Whole-Genome Sequences

Ten samples from AB, 3 samples from QC and 1 sample from BC yielded enough viral DNA and were directly submitted for WGS (Table 2). The size of the genomes varied between 150,118–153,648 kbps, with the smallest genome belonging to the BC isolate (CAN/BC-10-1122) due to a 3563-nucleotide deletion on the 5′ end of its genome. Interestingly deletion was also present in TCO-like and TCO vaccine sequences. The 14 Canadian ILTV sequences were aligned with 36 ILTV whole-genome sequences representing various geographical areas available in the public domain, which included TCO and CEO ILT vaccine sequences (Supplementary Table S2). The 14 Canadian ILTV sequences were deposited in the GenBank, and their accession numbers are given in Table 2.

### 3.3. Phylogenetic Analysis

**Figure 1:**
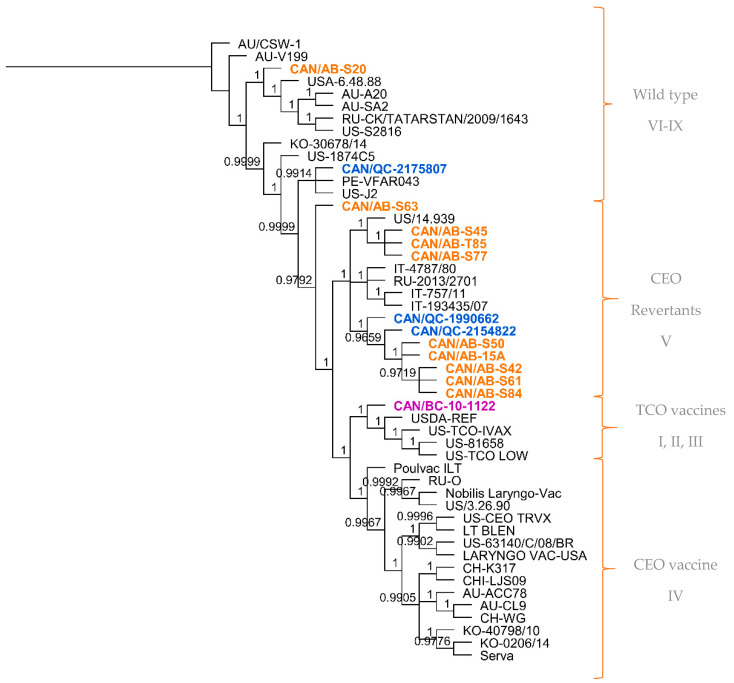
Phylogenetic tree of the full genome sequences of 50 ILTV strains from different geographical regions. Tree was generated using the Bayesian inference method (MrBayes, Geneious software). Posterior probability values are indicated as branch labels in the tree. Sequences highlighted in orange represent the Alberta ILTV isolates; in blue, the Quebec ILTV isolates and in purple is the ILTV isolate originated in British Columbia. Brackets and labels to the right of the tree separate and indicate the suggested genotype according to the clustering of the sequences in the phylogenetic tree.

Using the multiple sequence alignment of the 50 full genome sequences, a phylogenetic tree was generated. As shown in Figure 1, the first cluster mostly comprised wild-type ILTV isolates and virulent strains considered to be different from vaccines (genotype VI–IX), 3 of the 14 Canadian ILTV sequences clustered among this group (CAN/AB-S20, CAN/QC-2175807, CAN/AB-S63). The second cluster grouped ILTV isolates with CEO revertant viruses (genotype V), and 10 of the 14 Canadian ILTV sequences clustered with this group. The remaining Canadian ILTV sequence, represented by CAN/BC-10-1122, grouped within the cluster with the ILTV strains related to the TCO vaccines (genotype I, II, III). Additional information is shown in Supplementary Table S4. The last cluster included all the ILTV CEO commercial vaccine strains (Genotype IV), as well as European origin vaccine strain Serva, Chinese strains WG, K317, LJS09, Korean strain 40,798, US strains 63,140 and 3.26.90 and Australian strains CL9 and ACC78. None of the analyzed Canadian ILTV sequences clustered in this group.

**Figure 2:**
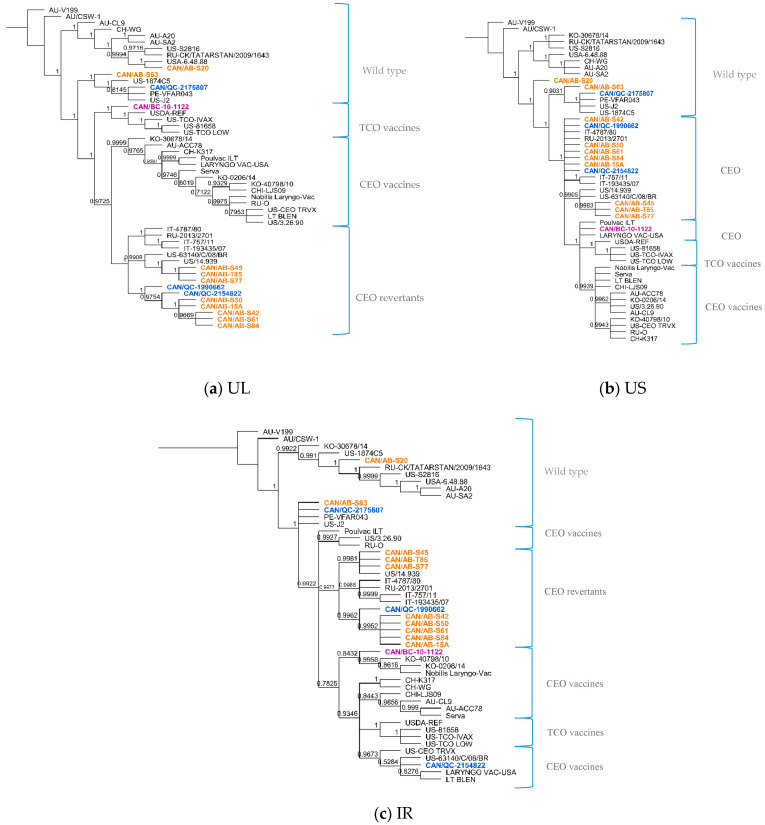
Phylogenetic analysis of 50 ILTV sequences using the unique long region (**a**), unique short region (**b**) and the internal repeat regions (**c**) using the Bayesian inference method (MrBayes, Geneious software). Sequences highlighted in orange represent the Alberta ILTV isolates; in blue, the Quebec ILTV isolates and in purple is the ILTV isolate originated in British Columbia. Brackets and labels to the right of the trees separate and indicate the suggested genotype according to the clustering of the sequences in the phylogenetic trees. Posterior probability values are indicated as branch labels in the tree.

Of the 10 Canadian ILTV isolates clustered as CEO revertants following whole-genome sequence analysis, it was interesting to find a high percent nucleotide identity between nine of these sequences (CAN/QC-1990662, CAN/AB-S61, CAN/AB-S50, CAN/AB-S42, CAN/AB-T85, CAN/AB-S45, CAN/AB-S77, CAN/AB-15A, CAN/AB-S84) and three vaccine strains: European Serva, Nobilis Laringovac^®^ (an attenuated ILTV Serva strain) and Poulvac ILT^®^ (uses the ILTV Salisbury strain). A comparative analysis was made between these nine ILTV Canadian sequences and the European Serva. On average, these nine sequences shared 99.9% nucleotide identity with 34 SNPs inside the coding sequence in 36 different ORFs (Table 3), including a codon insertion in ORF C, resulting in a frameshift in the amino acid sequence. Fourteen of these SNPs are synonymous, and 20 non-synonymous. The rest of the Canadian sequences, CAN/QC-2175807, CAN/AB-S63, CAN/BC-10-1122, and CAN/AB-S20, did not appear to be as closely related to the Serva strain or any of the latter mentioned vaccine strains. Additional phylogenetic analysis was conducted using three separate portions of the ILTV genome, the UL, US and IR regions (Figure 2a–c, respectively). Overall, the analyzed Canadian ILTV isolates clustered similarly compared to Figure 1 results, with the exception of isolate CAN/QC-2154822, which clustered with CEO vaccines and a US virulent strain on the IR phylogenetic tree.

The position within trees of sequence CAN/QC-2175807 remained constant along with VFAR043, J2 and Canadian CAN/AB-S63. A more detailed analysis of these sequences showed a close relationship among them. Between the first two mentioned strains, only 13 SNPs inside coding sequences differed between them, 7 of them synonymous and 6 non-synonymous. Between CAN/QC-2175807 and J2, only 10 SNPs inside coding sequences differed between them, 5 synonymous and 5 non-synonymous. The same comparison was made among sequences CAN/AB-S63, VFAR043 and J2. Between the first two sequences, 37 SNPs inside of coding sequences could be detected, 19 synonymous and 18 non-synonymous and between CAN/AB-63 and US strain J2, only 34 SNPs, 17 synonymous and 17 non-synonymous in 13 different coding sequences (UL2, US4, UL5, US5, UL6, UL9, UL23, UL25, UL27, UL32, UL38, UL39, UL41). CAN/QC-2154822 ILTV sequence, grouped with CEO vaccine strains (LT BLEN, LARYNGO-VAC and CEO TRVX) and a US strain 63140/C/08/BR in the IR region phylogenetic tree (Figure 2c), this Canadian isolate seemed to have more discrepancies with the rest of the sequences in the alignment. Hundred and forty-four (144) SNPs were identified inside coding sequences when compared to Serva, 21 SNPs synonymous and 123 non-synonymous, including 2 insertions, one in UL27, the other one in ORFC, and a total of 57 deletions, 36 of them concluding on frameshifts in the amino acid sequences in 21 coding regions. However, it was with Serva and Serva-like sequences that it was more closely related to. The Canadian ILTV isolate CAN/BC-10-1122, which clustered among TCO vaccines in the phylogenetic tree following the whole-genome sequence analysis, was found to cluster along with CEO vaccines in the phylogenetic trees of the US and IR regions (Figure 2b,c), showing a nucleotide percent identity of 100% with vaccine Poulvac ILT and Laryngo-vac vaccine sequences in the US multiple sequence alignment, and a 99.8% with Serva and Serva-like vaccine sequences in the IR region.

### 3.4. Recombination Analysis

Of the 14 Canadian ILTV whole-genome sequences examined for potential recombination events, CAN/BC-10-1122 was found to be a recombinant virus with vaccine strains as possible parental strains (Table 4). The first suggested parent is TCO vaccine LT-IVAX^®^, which shared an identity of 96.3% with the Canadian BC ILTV sequence. The analysis suggested two minor potential parents (i.e., sequences with a minor contribution to the genome of the suggested recombinant), US-CEO origin vaccine Poulvac ILT^®^, which shared 97.7% identity with the sequence CAN/BC-10-1122. CEO origin vaccine Nobilis Laryngo-vac^®^, which shared 97.4% identity with the Canadian isolate. Both CEO vaccine strains were almost identical, with 99.9% identity and only 19 nucleotide differences between them in the coding sequence. As shown in Table 4, the detection of recombination in the CAN/BC-10-1122 isolate using various methods was highly significant with very low *p* values.

A second recombination event was detected in sequence 6.48.88 of US origin. In this suggested recombination event, sequence CAN/AB-S20 was indicated as a minor parent (sequence with a minor contribution to the genome of the suggested recombinant), and vaccine SA2 was suggested as a potential major parent in this event (Table 4). As shown in Table 4, the detection of recombination in 6.48.88 ILTV isolate using various methods was highly significant with *p* values < 0.05.

Interestingly, RDP4 software pointed to another recombination event with sequence CAN/QC-2154822 as a major parent for Australian CL9 and minor parent A20. This recombination event had been previously described with Serva as a major parent for the CL9 isolate. After considering the background information on this isolate, belonging to Australia where vaccination with Serva is done with regularity, comparing and doing a visual verification of the alignment including the QC sequence, Serva and the rest of the isolates involved, this event was no longer taken into consideration. 

**Figure 3:**
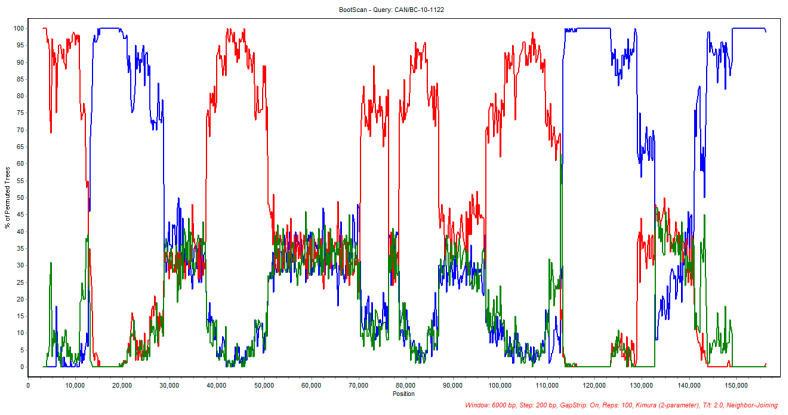
Bootscan analysis plot of CAN/BC-10-1122 with the suggested parental vaccine strain TCO LT-IVAX^®^ (red) and Poulvac ILT® strain (blue). US 1874C5 strain was used as control (green).

Both recombination events were originally revealed using RDP4 software, confirmed by RDP, GENCONV, MaxChi, Chimaera, SiScan and 3Seq methods [37,38,39,40,41,42]. To further confirm the RDP4 analysis results, a Bootscan analysis using the full genome sequence alignment done with MAFFT of the parental vaccine strains, suggested by RDP4 software, TCO LT-IVAX^®^ and Poulvac ILT^®^ was carried out. In this analysis, the US 1874C5ILTV strain was used as a control and CAN/BC10-1122 as the query sequence (Figure 3). Table 5 summarizes the percentage nucleotide identity of the BC ILTV isolate (BC-10-1122) with CEO Poulvac ILT^®^ and TCO LT-IVAX^®^ in different segments of the ILTV genome (1–15,393; 15,393–37,509; 37,509–113,748; 113,748–end) as indicated by bootscan analysis. It is noteworthy to mention that the same plot was obtained when using Nobilis Laryngo-vac^®^ as a minor parent in the Bootscan analysis.

**Figure 4:**
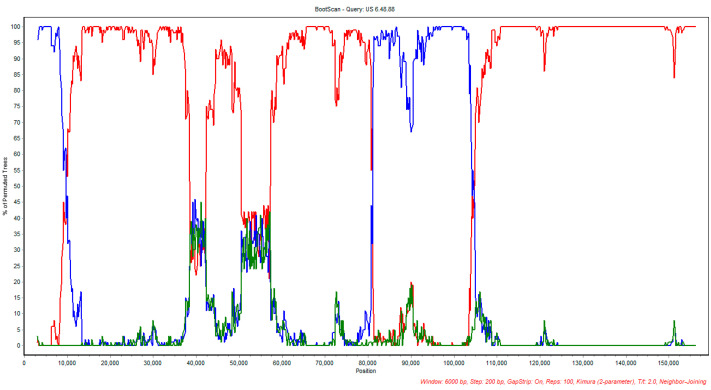
Bootscan analysis plot of CAN/AB-S20 with the suggested parental vaccine strain CAN/AB-S20 (blue) and SA2 (red). US 1874C5 strain was used as control (green).

The bootscan analysis also corroborated the recombination event involving CAN/AB-S20 ILTV and Australian SA2 as potential parental strains of US 6.48.88, as suggested by the RDP4 analysis; the results are illustrated in Figure 4. In this analysis, the US-TCO strain was used as a control and US 6.48.88 was used as the query sequence.

## 4. Discussion

The aim of the current study was to genetically characterize the ILTV isolates linked to ILT-positive cases in Canada using WGS. Among the highlights of this research was finding that most of the obtained Canadian sequences (*n* = 10) are genetically related to CEO vaccines. Our observation of CEO vaccine-related ILTV circulation in Canada is consistent with observations in other studies [1,21,43], and more recently, coinciding with findings on a study on the molecular characterization of circulating Western Canadian ILTV using *Iltovirus* specific genes, ORF a and b [26], where 84% ILTV isolates were characterized as genotype V, CEO revertants. It is noteworthy that some of the ILTV isolates belonging to AB and BC used in this study were also included in the previously mentioned study [26]. However, molecular characterization using partial ORF a and b genes did not classify any of the examined Canadian ILTV isolates as TCO vaccine-related. Instead, using partial ORF a and b genes, classified one of the isolates in this study, BC-10-1122 (considered to be TCO vaccine-related using WGS) as genotype IV, with the CEO vaccines, along with other two BC isolates [26].

In 1971, the BC poultry industry used three CEO attenuated vaccines for the vaccination of its flocks against ILT. A few years later, an attenuated vaccine (TCO origin) was also introduced to be used via eyedrop. During 1971–1973, the BC poultry industry encountered an ILT outbreak related to attenuated vaccines that involved 25–50% of the vaccinated flocks [3]. The introduction of live attenuated vaccines probably led to the circulation of the CEO vaccine virus in the province. The current practice of inadequate vaccination with the TCO vaccine (for example, administering the vaccine via drinking water, when it is indicated by the manufacturers for eyedrop administration only) may facilitate TCO vaccine virus reversion to virulence [12] and its circulation among the flocks in BC. The concurrent circulation of vaccine-related ILTV strains and wild-type ILTV probably aided in the recombination event found in the BC ILTV isolate.

In the case of AB, vaccination of commercial flocks is not a common practice, but it is done among backyard flocks. Based on flock history data (Table 1 and Supplementary Table S3), all Alberta-derived ILT samples in this study originated from noncommercial small back yard flocks. These flocks generally comprise different susceptible avian species of varying ages, and new birds are frequently introduced to the flock without adhering to proper biosecurity measures. Contact of these birds with other wild avian species and insects [44], which could be carriers of the disease, is also likely. Along with the transportation of show birds to various exhibitions and competitions, these set of conditions create an ideal situation for ILTV transmission and maintenance since biosecurity in such flocks cannot be maintained effectively [25,45].

It was also clear that Canada is not exempt from ILTV recombination events, as two of the 14 Canadian ILTV isolates (CAN/BC-10-1122 and CAN/AB-S20) appeared to be associated with recombination, and in both cases involving the presence of CEO or TCO live attenuated vaccine viruses, although vaccination with CEO attenuated vaccines is no longer recommended to be used in the Western part of Canada.

Highlighted by the results from the recombination event involving Australian vaccine strain SA2 and Canadian isolate CAN/AB-20, the results of this work are also in agreement with previous work suggesting a US origin for the Australian vaccine strain SA2, which could have later diversified and spread into different geographical areas [15]. This could explain its involvement in the later mentioned recombination event with the Canadian isolate. This view is based on sequence similarity of Canadian ILTV sequence CAN/AB-S20 with the Australian vaccine strains SA2 and A20, US field strains 6.48.88 and S2 816 and Russian strain RU/CK/TATARSTAN/2009/1043 and pointed out by the phylogenetic analysis results [15]. A US origin could also be the case for the Canadian isolate CAN/AB-S20. This event could have been facilitated too by the presence of wild species of migratory birds that could have played an important role in the spread and diversity of the viruses [7].

Similar to other viral families, recombination has been documented among different members of the *Herpesviridae* family, with many of the recombination events involving the use of attenuated vaccines [46,47,48,49,50]. In the case of ILTV, the attenuated CEO vaccines are usually involved in recombination events with more frequency than the TCO vaccines, which could be influenced by the higher degree of attenuation of the TCO vaccine strains. Recombination between attenuated ILT vaccines (either CEO or TCO) depends on a number of factors including, but not limited to, the quantity and ratio of the vaccines used [17]. However, naturally occurring ILTV recombination events are common, and our results are in agreement with these studies [16,22,23]. However, we have not investigated the consequence of these recombinant field ILTV isolates yet; it is important to know the frequency of these recombination events and whether they lead to increased virulence, as was recorded for other members of the *Herpesviridae* family such as the varicella-zoster virus (VZV) [50], HSV-1 and HSV-2) [50], and other ILTV recombinants [16,22,23]. Future work will be required to evaluate the pathogenicity and transmission potential of the Canadian recombinant ILTV isolates.

## 5. Conclusions

Using a WGS approach, we obtained the full genomes of 14 ILTVs of Canadian origin and detected naturally occurring recombination. The CEO vaccine viruses that circulate actively within chicken flocks in Canada added to the inadequate vaccination practices with live attenuated vaccines in some of the provinces, could favor recombination events between vaccine and wild-type viruses, as has been observed in the current study. Our findings add to the knowledge of ILTV evolution and challenges the practical implications of ILT control using live attenuated vaccines.

## Figures and Tables

**Table 1:** Relevant background information of the poultry farms from which the 14 Canadian infectious laryngotracheitis virus (ILTV) samples that yielded full genome sequences originated. Dash lines fill slots where information could not be obtained.

Sample ID	Province	Age (Weeks)	Breed	Type	Flock Size	ILT Vaccine Used	Morbidity # of Birds	Mortality # of Birds	Year
#1990662	Quebec	4 days	-	Broiler	19,700 females	No	-	-	2017
#2154822	Quebec	8	Ross	Broiler	6400	Recombinant	3000	400	2018
#2175807	Quebec	-	-	Broiler	17,000	No	-	527	2019
#10-1122	British Columbia	11	-	Layer	45,000	No	-	-	-
#15	Alberta	6	Heritage	Backyard	250	Yes **	10	10	2014
#20	Alberta	40	Mille fleur	Backyard	150	No	4	4	2015
#42	Alberta	60	Heritage	Backyard	56	No	22	4	2016
#45	Alberta	24	Barnevelder	Backyard	50	No	4	4	2016
#50	Alberta	10	PRS cross *	Backyard	475	No	400	40	2016
#61	Alberta	96	Heritage	Backyard	50	No	15	15	2017
#63	Alberta	6	Heritage mixed	Backyard	150	No	5	5	2017
#77	Alberta	80	Heritage	Backyard	150	No	7	4	2017
#84	Alberta	22	Heritage	Backyard	50	No	5	0	2017
#85	Alberta	40	Heritage	Backyard	120	No	80	80	2017

* PRS cross = Plymouth-Rhode Island-Sussex cross; ** Further details on the type of vaccine applied could not be gathered.

**Table 2:** Canadian ILTV full genome sequences (*n* = 14).

Isolate	Genome Length	Province	Total Reads	Mapped Reads	Virus Isolation	Accession #
CAN/AB-15A	153,648	Alberta	5,650,374	9220	LMH cells	MT797239
CAN/AB-S20	152,695	Alberta	3,630,632	18,054	LMH cells	MT797240
CAN/AB-S42	153,469	Alberta	3,128,494	75,084	LMH cells	MT797241
CAN/AB-S45	153,630	Alberta	4,144,190	55,821	LMH cells	MT797242
CAN/AB-S50	153,641	Alberta	2,394,680	41,448	LMH cells	MT797243
CAN/AB-S61	153,643	Alberta	3,603,066	15,064	LMH cells	MT797244
CAN/AB-S63	152,703	Alberta	2,660,390	9072	LMH cells	MT797245
CAN/AB-S77	153,633	Alberta	3,245,498	17,863	LMH cells	MT797246
CAN/AB-S84	153,643	Alberta	3,090,720	9735	LMH cells	MT797247
CAN/AB-T85	153,631	Alberta	2,504,122	12,822	LMH cells	MT797248
CAN/BC-10-1122	150,118	British Columbia	2,404,772	502,040	LMH cells	MT797249
CAN/QC-1990662	153,598	Quebec	4,669,964	67,376	CAM	MT797250
CAN/QC-2154822	151,326	Quebec	4,233,878	9055	CAM	MT797251
CAN/QC-2175807	153,468	Quebec	8,493,852	50,518	CAM	MT797252

**Table 3:** Single nucleotide polymorphisms (SNPs) of the nine Canadian ILTV sequences from the Alberta (CAN/AB-S61, CAN/AB-S50, CAN/AB-S42, CAN/AB-T85, CAN/AB-S45, CAN/AB-S77, CAN/AB-15A, CAN/AB-S84) and Quebec (CAN/QC-1990662) provinces using the vaccine strain Serva as a reference sequence. Asterisks (*) represent all nine Canadian sequences. RDR = ribonucleoside-diphosphate reductase, DUTN = deoxyuridine 5′-triphosphate nucleotidohydrolase.

Gene	Protein	CDS Position	Nucleotide Change	Amino Acid Change	Sequence
US7	Envelope glycoprotein	57	C → T		CAN/AB-45, 84 and 77
UL39	RDR large subunit	58	G → A	D → N	CAN/AB-45, 84 and 77
UL35	Large tegument protein	89	C → T	T → I	CAN/AB-45, 84 and 77
UL1	Uracil-DNA glycosylase	121	C → T	D → N	CAN/AB-15, 42, 50, 61, 84 and CAN/QC-1990662
UL10	Envelope gM	124	T → C	T → A	*
US10	Virion protein	128	A → G	D → G	CAN/AB-45, 84 and 77
US10		128	T → C	D → G	CAN/AB-45, 84 and 77
UL1		161	T → G	Q → P	*
UL27	Envelope gB	347	A → G	V → A	*
ORFB	ORF B protein	352	A → C		*
ORFA	ORF A protein	360	A → C		*
UL49	Envelope gN	378	T → C		CAN/AB -15, 42, 50, 61& 84
ORFE	ORF E protein	398	C → G	G → A	*
UL50	DUTN	453	A → G		*
US8	Envelope gE	629	A → G	K → R	*
UL46	Putative viral tegument protein	849	A → G		*
UL21	Tegument protein	924	C → T		CAN/AB -15, 42, 50, 61& 84
UL5	DNA replication helicase	1027	A → G	K → E	*
US6	Envelope gD	1164	C → T		CAN/AB-45, 84 and 77
US3	Protein Kinase	1200	A → G		*
UL44	Envelope gC	1201	T → C		CAN/AB -15, 42, 50, 61& 84
UL9	DNA replication origin-binding helicase	1428	G → C	Q → H	CAN/AB -15, 42, 50, 61& 84
ORFF	ORF F protein	1878	T → C		CAN/AB-15, 42, 50, 61, 84 and CAN/QC-1990662
ORFF		1883	C → A	S → Y	CAN/AB-45, 84 and 77
ORFF		1899	CT → TA	GS → GT	CAN/AB-15, 42, 50, 61, 84 and CAN/QC-1990662
UL28	Tripartite terminase subunit 1	1913	A → G	V → A	*
UL27	Envelope gB	1931	A → G	I → T	*
UL52	Helicase-primase primase subunit	2232	A → T	F → L	CAN/AB -15, 42, 50, 61& 84
UL52		2256	G → A		CAN/AB -15, 42, 50, 61& 84
UL52		2325	C → T		CAN/AB -15, 42, 50, 61& 84
ICP4	Major viral transcription factor	2342	T → C	H → R	*
ICP4		2342	A → G	H → R	*
UL36	Large tegument protein	2449	G → A	R → C	CAN/AB-15, 42, 50, 61, 84 and CAN/QC-1990662
UL36		4040	C → T	R → H	*
ICP4	Major viral transcription factor	4281	C → T		*
ICP4		4281	G → A		*
UL36	Large tegument protein	7677	T → C		*
UL36		8349	C → A		CAN/AB-45, 84 and 77

**Table 4:** Recombination signals involving Canadian ILTV isolates, recombination analysis carried out on Recombination Detection Program (RDP4) software.

Potential Recombinant	Potential Major Parent	Potential Minor Parent	Detection Methods	*p*-Values	Position of Recombination Breaking Points
BC-10-1122	LT-IVAX	Poulvac ILT Nobilis Laryngo-vac	RDP GENECONV MaxChi Chimaera SiScan 3Seq	2.379 × 10^−6^; 3.356 × 10^−9^ * 1.457 × 10^−5^; 3.362 × 10^−8^ * 3.060 × 10^−6^; 6.860 × 10^−8^ * 1.518 × 10^−6^; 3.337 × 10^−8^ * 1.476 × 10^−5^; 1.054 × 10^−7^ * 1.194 × 10^−7^; 2.190 × 10^−3^ *	15,393-UL52 37,509-UL26 113,748-ICP4
6.48.88	SA2	CAN/AB-S20	RDP GENCONV MaxChi Chimaera SiScan 3Seq	5.375 × 10^−^^20^ 1.864 × 10^−^^18^ 2.159 × 10^−^^10^ 1.463 × 10^−^^10^ 2.379 × 10^−11^	9652-ORF F 81,152-UL 19 104,647-UL 5

* *p*-value attributed to recombination event including LT IVAX and Nobilis Laryngo-vac as a potential minor parent.

**Table 5:** Percentages of nucleotide identity of vaccines CEO-Poulvac ILT^®^ and TCO-LT-IVAX^®^with British Columbia ILTV isolate (BC-10-1122) indicated by bootscan.

	1–15,393	15,393–37,509	37,509–113,748	113,748–End
Poulvac ILT	77.09%	99.98%	99.84%	99.52%
TCO_IVAX	99.41%	99.91%	99.83%	87.99%

## Supplementary Materials

The following are available online at https://www.mdpi.com/1999-4915/12/11/1302/s1, Table S1. Depth variation along the genome of the 14 ILTV Canadian sequences, Table S2. The representative ILTV whole genome sequences (*n* = 36) that were obtained from public domain and their genome length and Genbank accession numbers for the purpose of aligning with the Canadian ILTV whole genome sequences, Table S3. Clinicopathological history of the ILTV infected chickens where the 14 Canadian ILTV samples that yielded full genome sequences originated, Table S4. Proposed genotype for the 14 Canadian ILTV sequences adressed in this study, and average percent identity with the rest of the sequences belonging in the corresponding groups.
