# Supplementary material for: Analysis of Whole-Genome Sequences of Infectious laryngotracheitis Virus Isolates from Poultry Flocks in Canada: Evidence of Recombination
Source: Viruses. 2020 Nov 12;12(11):1302. doi: 10.3390/v12111302 (PMC7696358; doi:10.3390/v12111302)
Supplement: Supplementary file 1 [file viruses-12-01302-s001.pdf]

Supplementary Materials:

Table S1. Depth variation along the genome of the 14 ILTV Canadian sequences.

Sequence	GenBank Reference Accession #	Reference Length	Consensus Length	Minimum Coverage	Maximum Coverage	Median Coverage	Average Coverage	Positions with Coverage Below 5
CAN/QC-1990662	VHTSL sequence*	153629	153590	2	178	67	69,43	39
CAN/QC-2154822	MF417811	151327	151297	1	45	9	9,54	30
CAN/QC-2175807	MG775218	152701	152535	1	120	34	35,44	166
CAN/BC-10-1122	VHTSL sequence	151495	151452	1	1295	652	622,34	43
CAN/AB-S15	KP677885	153653	151802	1	35	10	10.64	1851
CAN/AB-S20	JX466898	153630	153169	1	91	25	25.64	461
CAN/AB-S42	KP677885	153653	153469	1	251	119	118.69	184
CAN/AB-S45	MF417811	153629	153539	1	208	81	81.87	90
CAN/AB-S50	KP677885	153653	153389	1	145	67	66.02	264
CAN/AB-S61	KP677885	153653	153005	1	58	23	23.24	648
CAN/AB-S63	MG775218	152701	151703	1	46	15	15.14	998
CAN/AB-S77	MF417811	153629	153133	1	77	26	26.18	496
CAN/AB-S84	KP677885	153662	151963	1	46	14	14.63	1699
CAN/AB-S85	MF417811	153629	152795	1	59	22	22.13	834

*Veterinary high-throughput sequencing laboratory ILTV sequence.

Table S2. The representative ILTV whole genome sequences (n = 36) that were obtained from public domain and their genome length and Genbank accession numbers for the purpose of aligning with the Canadian ILTV whole genome sequences.

Strain	Country	Genome Length	Accession Number
TCO LOW	USA	155,465	JN580315
TCO IVAX	USA	155,465	JN580312
S2816	USA	154,001	MF417807
J2	USA	153,711	MF417808
USDA REF	USA	151,756	JN542534
CEO TRVX	USA	153,647	JN580313
6.48.88	USA	154,022	MF417810
81658	USA	150,335	JN542535
63140/C/08/BR	USA	153,633	JN542536
1874C5	USA	149,682	JN542533
3.26.90	USA	153,655	MF417809
14.939	USA	153,629	MF417811
SERVA	Europe	152,630	HQ630064
O	Russia	153,634	KU128407
CK/TATARSTAN/2009/1643	Russia	153,933	MF405079
2013/2701	Russia	153,634	MF405080
VFAR043	Peru	153,634	MG775218
NOBILIS LARYNGOVAC	USA	152,701	KP677881
LT BLEN	USA	153,623	JQ083493
LARYNGOVAC USA	USA	153,624	JQ083494
40798/10	Korea	153,649	MH937566
30678	Korea	153,659	MH937565
0206/14	Korea	153,645	MH937564
POLUVAC ILT	Italy	153,650	KP677882
193435/07	Italy	153,662	KP677883
4787/80	Italy	153,653	KP677885
757/11	Italy	153,662	KP677884
WG	China	153,505	JX458823
K317	China	153,639	JX458824
LJS09	China	153,201	JX458822
V199	Australia	153,630	JX646898
SA2	Australia	152,975	JN596962
CL9	Australia	152,635	JN804827
ACC78	Australia	152,632	JN804826
A20	Australia	152,978	JN596963
CSW-1	Australia	151,671	JX646899

Table S3. Clinicopathological history of the ILTV infected chickens where the 14 Canadian ILTV samples that yielded full genome sequences originated. Dash lines fill slots where information could not be obtained.

Sample ID	Province of Origin	Clinical Signs	Gross Lesions	Histopathological Lesions
#1990662	Quebec	Dyspnea and respiratory rales	-	-
#2154822	Quebec	Dyspnea and respiratory rales	-	-
#2175807	Quebec	Conjunctivitis and respiratory rales	Hemorrhagic tracheitis	-
#10-1122	British Columbia	-	-	-
#15	Alberta	Lethargy, extended neck respiration with respiratory rales and gasping	Hemorrhagic tracheitis	Lymphoplasmacytic laryngotracheitis with syncytial cells and intranuclear viral inclusions
#20	Alberta	Periorbital inflammation, dyspnea and sudden death	Catarrhal laryngotracheitis	Lymphoplasmacytic laryngotracheitis with syncytial cells and intranuclear viral inclusions
#42	Alberta	Gasping and dyspnea with respiratory rales	Fibrinohemorrhagic and necrotizing laryngotracheitis	Fibrinohemorrhagic and necrotizing, and lymphoplasmacytic and heterophilic laryngotracheitis with syncytial cells and intranuclear viral inclusions
#45	Alberta	Periorbital inflammation, ocular secretion and conjunctivitis	Fibrinonecrotizing laryngotracheitis	Fibrino necrotizing, lymphoplasmacytic and heterophilic laryngotracheitis with syncytial cells and intranuclear viral inclusions
#50	Alberta	Dyspnea and extended neck respiration with respiratory rales	Fibrinohemorrhagic and necrotizing laryngotracheitis	Fibrinohemorrhagic and necrotizing, and lymphoplasmacytic and heterophilic laryngotracheitis with syncytial cells and intranuclear viral inclusions
#61	Alberta	Breathing with respiratory rales	Fibrinonecrotizing laryngotracheitis	Fibrinohemorrhagic and necrotizing, and lymphoplasmacytic and heterophilic laryngotracheitis with syncytial cells and intranuclear viral inclusions

#63	Alberta	Nasal and ocular secretions and facial edema, depression	Fibrinohemorrhagic laryngotracheitis	Lymphoplasmacytic laryngotracheitis with syncytial cells and intranuclear viral inclusions
#77	Alberta	Periorbital edema and extended neck respiration and gasping	Catarrhal and necro-hemorrhagic laryngotracheitis	Catarrhal, fibrinohemorrhagic and necrotizing, lymphoplasmacytic and heterophilic laryngotracheitis with syncytial cells and intranuclear viral inclusions
#84	Alberta	Periorbital edema and sneezing	Fibrinonecrotizing laryngotracheitis	Lymphoplasmacytic and heterophilic fibrinonecrotic laryngotracheitis
#85	Alberta	Lethargy and gasping	Fibrinonecrotizing laryngotracheitis	Fibrinohemorrhagic and necrotizing, lymphoplasmacytic and heterophilic laryngotracheitis

Table S4. Proposed genotype for the 14 Canadian ILTV sequences adressed in this study, and average % identity with the rest of the sequences belonging in the corresponding groups.

Canadian ILTV Sequences	Genotype	Average % Identity	ILTV sequences Belonging to this Genotype
CAN/QC-2175807<br>CAN/AB-S63<br>CAN/AB-S20	Wild-type (VI- IX)	99.7%	VFAR043, VI99, CSW-1, J2, 1874C5 and 6.48.88, CK/TATARSTAN, Ko-30678/14
CAN/QC-1990662<br>CAN/AB-S61<br>CAN/AB-S50<br>CAN/AB-S42<br>CAN/AB-T85<br>CAN/AB-S45<br>CAN/AB-S77<br>CAN/AB-15A<br>CAN/AB-S84<br>CAN/QC-2154822	CEO revertant (V)	99.8%	US/14.939, IT-4787/80, RU-2013/2701, IT757/11, IT-193435/07
CAN/BC-10-1122	TCO vaccine (I,II,III)	97.7%	TCO-IVAX, TCO-Low, USDA ref, US-81658
